# Monocytes and cervical ripening: a narrative review of prolonged labor pathophysiology

**DOI:** 10.1097/MS9.0000000000003004

**Published:** 2025-05-21

**Authors:** Emmanuel Ifeanyi Obeagu, Salma Abdi Mahmoud

**Affiliations:** a Department of Biomedical and Laboratory Science, Africa University, Mutare, Zimbabwe and; b Department of Obstetrics and Gynaecology, School of Health and Medical Sciences, The State University of Zanzibar, Zanzibar, Tanzania

**Keywords:** cervical ripening, cytokines, inflammation, monocytes, prolonged labor

## Abstract

Prolonged labor, a major obstetric complication, is often linked to inadequate cervical ripening, which hinders labor progression. The process of cervical ripening is governed by complex hormonal and immune-mediated mechanisms, with monocytes playing a central role. These immune cells infiltrate the cervix and differentiate into macrophages, releasing cytokines and proteases that are essential for extracellular matrix (ECM) remodeling, cervical softening, and dilation. However, in prolonged labor, an imbalance in monocyte activity may impede normal cervical ripening, contributing to stalled labor and increased risk of maternal and neonatal complications. Monocytes are critical to the inflammatory response that initiates cervical remodeling during labor. Upon recruitment to the cervix, monocytes release inflammatory cytokines like interleukin (IL)-1, IL-6, and tumor necrosis factor-alpha, which activate matrix metalloproteinases to degrade collagen and ECM proteins, facilitating cervical effacement and dilation. Dysregulated monocyte recruitment and prolonged inflammation, however, may lead to ineffective cervix remodeling, preventing labor from progressing efficiently. Furthermore, these immune responses can influence uterine contractility, either promoting or inhibiting uterine contractions, which further complicates the pathophysiology of prolonged labor.

## Introduction

Prolonged labor, often defined as labor that lasts more than 20 hours in nulliparous women or 14 hours in multiparous women, is a significant obstetric complication that can lead to maternal and neonatal morbidity and mortality^[1]^. It is often characterized by a failure of the cervix to dilate or efface at the expected rate during the first and second stages of labor^[2]^. Several factors contribute to prolonged labor, including maternal pelvic abnormalities, uterine dysfunction, fetal malpresentation, and psychological factors^[3]^. However, one of the most critical contributors to the progression of labor is the process of cervical ripening, which is essential for labor to progress smoothly^[4]^. Inadequate cervical ripening, often referred to as “cervical dystocia,” is frequently observed in prolonged labor and can impede vaginal delivery, leading to a higher risk of requiring cesarean section^[5]^. Cervical ripening is a dynamic process that involves the softening, thinning, and dilation of the cervix in preparation for labor^[6]^. The cervix must undergo significant remodeling, which is facilitated by a variety of biochemical, hormonal, and immune factors^[7]^. During pregnancy, the cervix is composed mainly of collagen and other extracellular matrix (ECM) proteins, providing it with strength and rigidity to maintain the integrity of the uterine environment. As labor approaches, the cervix undergoes structural and biochemical changes that reduce its firmness, allowing for progressive dilation during contractions^[8]^. This remodeling process is influenced by a coordinated interaction between inflammatory cells, cytokines, proteases, and other mediators, among which monocytes play a pivotal role^[9]^.

**HIGHLIGHTS**

- *Immune involvement*: Highlights monocytes’ pivotal role in cervical ripening through inflammatory mediators and tissue remodeling.
- *Prolonged labor insights*: Links altered monocyte function with disrupted cervical maturation, contributing to prolonged labor pathophysiology.
- *Cytokine regulation*: Reviews monocyte-driven cytokine profiles influencing collagen breakdown and extracellular matrix remodeling in cervical ripening.
- *Therapeutic potential*: Explores targeting monocyte pathways as potential interventions to address prolonged labor complications.
- *Research gaps*: Identifies the need for studies on monocyte phenotypes and signaling pathways in cervical ripening and labor outcomes.

Monocytes, a type of white blood cell integral to the innate immune system, are recruited to the cervix during labor in response to inflammatory signals^[10]^. These cells differentiate into macrophages upon reaching the cervix and release a variety of cytokines, growth factors, and matrix metalloproteinases (MMPs)^[11]^. These substances are essential for the degradation of collagen and other ECM components, facilitating cervical softening. Monocytes also play a role in regulating uterine contractility by releasing pro-inflammatory cytokines, such as interleukin (IL)-6, tumor necrosis factor-alpha (TNF-α), and IL-1, which stimulate uterine smooth muscle cells to contract^[12]^. This inflammation-driven contraction process is essential for the progression of labor, as it helps to dilate the cervix and move the fetus down the birth canal^[13]^. However, when this inflammatory process becomes dysregulated, it may contribute to prolonged labor, as the cervix may fail to soften and dilate adequately^[14]^. The interplay between monocytes, cytokines, and other inflammatory mediators is central to the pathophysiology of cervical ripening^[15]^. Under normal circumstances, the recruitment of monocytes to the cervix is a tightly regulated process, ensuring that inflammation occurs at the appropriate time and intensity^[16]^. However, in prolonged labor, this process may become dysregulated, leading to excessive inflammation or insufficient cervical remodeling^[3]^. In some cases, chronic inflammation may result in an imbalance of pro- and anti-inflammatory cytokines, disrupting the normal physiological changes needed for cervical dilation^[6]^. This immune dysfunction can contribute to uterine inertia, where contractions are either insufficient or ineffective in facilitating labor progression^[9]^.

Moreover, the prolonged activation of monocytes and macrophages in the cervix can lead to the excessive release of proteases, such as MMPs, which degrade the ECM and may weaken the cervix too much or disrupt the normal tissue integrity^[17]^. This pathological remodeling of the cervix may impede its ability to fully efface and dilate during labor, ultimately leading to the failure of labor to progress^[18]^. In other cases, the inflammatory response may lead to a state of immune tolerance, where the cervix becomes unresponsive to the inflammatory cues necessary for labor progression^[16]^. This failure of cervical ripening is one of the key mechanisms by which prolonged labor can occur. Additionally, the role of monocytes in prolonged labor extends beyond their involvement in cervical ripening^[19]^. These immune cells are also implicated in regulating uterine contractility, a process that is critical for labor progression. Pro-inflammatory cytokines released by monocytes, such as IL-1, IL-6, and TNF-α, can stimulate uterine smooth muscle cells to initiate contractions^[20]^. However, when monocyte activation becomes dysregulated, it can lead to ineffective or irregular uterine contractions, which may further contribute to stalled labor^[21]^. This dysfunction in uterine contractility is often observed in cases of prolonged labor and is one of the key challenges in managing women with this condition^[22]^. The inflammatory mediators released by monocytes not only influence cervical and uterine function but also play a role in coordinating the overall labor process^[23]^. In a healthy labor progression, the immune response leads to a balanced inflammatory cascade that facilitates cervical ripening, uterine contractions, and ultimately the expulsion of the fetus^[12]^. However, in prolonged labor, this balance can be disrupted, leading to a failure of one or more components of labor to progress at the appropriate pace. Recent advances in the study of immune cells and inflammation in labor have highlighted the potential therapeutic applications of modulating the monocyte-driven inflammatory response^[8]^. Targeting specific inflammatory pathways, such as cytokine signaling or monocyte recruitment, may offer new opportunities to enhance cervical ripening and uterine contractility in women with prolonged labor^[9]^. For example, the use of anti-inflammatory drugs or cytokine inhibitors may help to regulate the inflammatory response, promoting cervical dilation and improving the efficiency of uterine contractions^[2]^. Additionally, therapies that aim to stimulate or modulate monocyte function could help to accelerate labor progression, reducing the need for invasive interventions such as cesarean sections^[1]^.

## Aim

The aim of this review article is to explore the role of monocytes in the pathophysiology of prolonged labor, with a specific focus on their involvement in cervical ripening, uterine contractions, and the inflammatory environment of the cervix.

## Rationale of the review

Prolonged labor remains a significant clinical challenge, contributing to adverse maternal and neonatal outcomes, including increased risk of cesarean delivery, maternal morbidity, and fetal distress. Despite extensive research into its causes, the precise mechanisms underlying prolonged labor remain poorly understood, particularly the role of the immune system in cervical ripening and uterine function. The involvement of monocytes and their derived cytokines in the inflammatory processes that govern cervical remodeling, uterine contractility, and overall labor progression has only recently begun to attract attention. This emerging understanding of monocyte function in the context of labor presents an opportunity to enhance current clinical practices and identify novel therapeutic targets for the management of prolonged labor.

The rationale for this review is based on the growing body of evidence suggesting that immune cells, especially monocytes, play a central role in the inflammatory cascade that regulates cervical ripening and uterine contractions. Dysregulation of this immune response may impair the normal progression of labor, contributing to prolonged labor and its associated complications. By synthesizing current research on monocyte activity during labor, this review aims to bridge knowledge gaps regarding the pathophysiology of prolonged labor and provide insights into how immune modulation could be utilized as a therapeutic strategy. Moreover, the review seeks to investigate the potential of targeting specific cytokines or immune pathways to improve labor progression, reduce the need for invasive interventions, and optimize maternal and fetal health outcomes. In light of the increasing prevalence of interventions such as cesarean sections, it is crucial to identify alternative therapeutic strategies that can facilitate natural labor progression. This review seeks to contribute to the clinical understanding of prolonged labor by highlighting the role of immune dysfunction and proposing potential interventions, which could lead to improved management strategies. Ultimately, a deeper understanding of the molecular mechanisms involved in labor progression will inform the development of more effective, targeted therapies that could significantly reduce the burden of prolonged labor worldwide.

### Review methodology

This narrative review was conducted to explore the role of monocytes in the pathophysiology of prolonged labor, particularly their involvement in cervical ripening, uterine contractions, and inflammation. The methodology involved a systematic process of identifying, selecting, and analyzing relevant studies to synthesize existing knowledge and identify gaps in current research.

- *Literature search*: A comprehensive literature search was performed using databases such as PubMed, Scopus, and Google Scholar. The search included studies published between 2000 and 2024. Keywords used for the search included “monocytes,” “cervical ripening,” “prolonged labor,” “inflammation,” “cytokines,” and “uterine contractions.”
- *Study selection*: Eligible studies were selected based on the relevance to the review topic, including original research articles, reviews, and clinical studies. The inclusion criteria focused on studies that investigated the role of monocytes and inflammatory cytokines in cervical ripening, labor progression, and the pathophysiology of prolonged labor. Studies with clear descriptions of immune mechanisms in labor, as well as those examining potential therapeutic interventions, were prioritized.
- *Data extraction*: Key data were extracted from the selected studies, including information on the immune pathways involved in cervical ripening, the function of monocytes during labor, and the impact of immune modulation on labor outcomes. Studies examining the effects of cytokine inhibitors, oxytocin, prostaglandins, and other interventions on monocyte activity were also reviewed.
- *Critical analysis*: The articles were critically analyzed for the quality of the study design, methodology, and relevance to the review topic. The review process involved synthesizing findings on monocyte activation and recruitment, inflammatory markers associated with labor, and therapeutic strategies aimed at improving labor progression. Key themes were identified to provide a cohesive understanding of how immune responses contribute to prolonged labor.
- *Synthesis of findings*: Based on the critical analysis, the findings were synthesized into key themes, which include monocyte recruitment and activation, cytokine production, dysregulation in prolonged labor, and potential therapeutic interventions. The synthesis of these findings offers a comprehensive overview of the role of monocytes in prolonged labor and potential strategies for managing labor progression.

### Monocyte recruitment and activation in cervical ripening

Monocyte recruitment and activation in cervical ripening are essential processes in the physiological preparation of the cervix for labor. Monocytes, as a part of the innate immune system, play a pivotal role in mediating the inflammatory response required for cervical remodeling^[24]^. The process of cervical ripening, which involves the softening, thinning, and dilation of the cervix, is closely regulated by inflammatory mediators, including cytokines, proteases, and immune cells, with monocytes being crucial in the initial stages of cervical remodeling^[25]^. In response to labor-related signals, these cells are recruited to the cervix, where they undergo activation and differentiation, contributing to the complex tissue changes necessary for effective labor progression^[26]^. The recruitment of monocytes to the cervix is primarily driven by pro-inflammatory cytokines and chemokines that are upregulated in response to mechanical, hormonal, or biochemical signals from the uterus and cervix^[27]^. Key cytokines such as IL-1, IL-6, and TNF-α are involved in the recruitment of monocytes from the bloodstream to the cervix^[28]^. Additionally, the chemokine CCL2 (monocyte chemoattractant protein-1, MCP-1) plays a critical role in guiding monocytes toward the cervix, where they adhere to the endothelial cells of blood vessels and transmigrate into the tissue^[29]^. Once monocytes infiltrate the cervix, they undergo differentiation into macrophages, a process that is essential for their subsequent activation and function during cervical ripening^[30]^.

**Figure 1.**
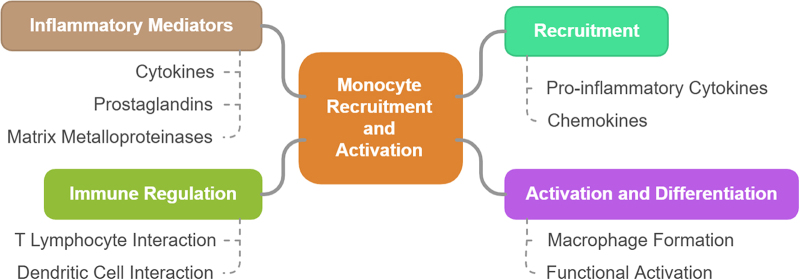
Schematic pathophysiology of monocyte recruitment and activation in cervical ripening.

Once within the cervix, the activated monocytes (now macrophages) release a variety of inflammatory mediators, including cytokines, prostaglandins, and MMPs^[31]^. These molecules are integral to the breakdown of the ECM, facilitating cervical softening and dilation. MMPs, in particular, are responsible for degrading collagen and other ECM components that provide structural integrity to the cervix, allowing it to soften and open in preparation for the passage of the fetus^[32-34]^. This remodeling process is further amplified by the release of additional pro-inflammatory cytokines, such as IL-1, IL-6, and TNF-α, which promote uterine contractility and enhance cervical effacement^[35]^. Furthermore, monocytes’ activation in cervical ripening is not limited to their secretion of pro-inflammatory mediators. These cells also contribute to the regulation of local immune responses in the cervix^[36]^. Their interactions with other immune cells, such as T lymphocytes and dendritic cells, further modulate the inflammatory environment, ensuring the appropriate balance between pro-inflammatory and anti-inflammatory signals^[37]^. Dysregulation of these interactions can lead to excessive inflammation, which may result in impaired cervical ripening, potentially contributing to prolonged labor^[38]^. On the other hand, inadequate monocyte activation or insufficient inflammatory response may hinder the necessary tissue changes in the cervix, delaying the onset of labor^[39]^. The process of monocyte recruitment and activation in cervical ripening highlights the intricate connection between the immune system and the mechanical processes that govern labor^[40]^ as shown in Fig. 1. The timely and controlled activation of monocytes ensures that cervical remodeling occurs in response to both hormonal and inflammatory signals, facilitating efficient labor progression^[31,35]^. However, when this process becomes dysregulated, it can lead to pathological labor outcomes, such as prolonged labor, uterine dysfunction, or failure to progress^[29]^. Understanding the molecular pathways that control monocyte recruitment, differentiation, and activation could lead to novel therapeutic strategies aimed at modulating the inflammatory response in women with labor complications, thus improving maternal and neonatal outcomes^[20,23,26]^. Figure 1 shows the schematic pathophysiology of monocyte recruitment and activation in cervical ripening.

### Role of monocyte-derived cytokines in cervical ripening

Monocyte-derived cytokines play a central role in cervical ripening, a crucial process in preparation for labor^[41]^. These cytokines, produced by activated monocytes and their differentiated macrophage counterparts, orchestrate the inflammatory response required for cervical remodeling, which involves softening, thinning, and dilation of the cervix^[35]^. As the cervix transitions from a firm, closed structure to one that is soft and dilated to allow for fetal passage, a complex interplay of cytokines, proteases, and other inflammatory mediators ensures that the tissue changes necessary for labor to progress efficiently occur^[25]^. Monocyte-derived cytokines, including IL-1, IL-6, TNF-α, and IL-8, are integral to this process, acting to mediate and amplify the inflammatory cascade that triggers cervical ripening^[42]^. One of the primary cytokines involved in cervical ripening is IL-1, which is produced by monocytes in response to inflammatory signals^[43]^. IL-1 promotes the production of other cytokines, such as IL-6 and TNF-α, and plays a critical role in initiating the inflammatory process necessary for cervical remodeling^[44]^. IL-1 enhances the expression of MMPs, which are enzymes responsible for the degradation of collagen and other components of the ECM in the cervix^[45]^. This ECM degradation is essential for the softening and thinning of the cervix, which allows for dilation during labor. In addition, IL-1 has been shown to increase prostaglandin production, further promoting cervical ripening and uterine contractions, both necessary for the progression of labor^[46]^.

IL-6, another key cytokine derived from monocytes, plays a multifaceted role in cervical ripening and labor initiation. IL-6 is a potent pro-inflammatory cytokine that contributes to immune responses, but it also has direct effects on uterine contractility and cervical remodeling^[47]^. IL-6 promotes the synthesis of MMPs, which facilitate the breakdown of ECM components, and regulates the expression of other cytokines involved in the inflammatory cascade^[45]^. Additionally, IL-6 contributes to the systemic inflammatory response that characterizes labor, signaling the onset of parturition and influencing uterine smooth muscle contraction^[48]^. This cytokine also aids in the modulation of prostaglandin synthesis, enhancing uterine contractions and facilitating cervical effacement and dilation. TNF-α is another critical monocyte-derived cytokine that plays an essential role in cervical ripening^[49]^. TNF-α is involved in both local and systemic inflammatory responses and is known to induce the expression of MMPs in the cervix, leading to the breakdown of collagen and other ECM components^[50]^. This activity promotes the softening and remodeling of the cervix, essential for labor progression. TNF-α also enhances the production of prostaglandins, which further contribute to cervical dilation and uterine contractions^[50]^. In addition, TNF-α acts as a potent mediator of uterine contractions, facilitating the expulsion of the fetus during labor. The upregulation of TNF-α at the site of the cervix during labor initiation underscores its significant role in facilitating the physiological changes necessary for vaginal delivery^[51]^.

IL-8, a chemokine released by monocytes, also plays a critical role in cervical ripening. IL-8 is primarily known for its role in recruiting neutrophils to sites of infection and injury, but it also has important effects on the cervix^[47]^. It contributes to cervical remodeling by increasing the expression of MMPs and promoting the breakdown of the ECM. IL-8 also acts as a chemotactic factor, recruiting additional immune cells, including neutrophils and other monocytes, to the cervix^[41]^. This influx of immune cells intensifies the inflammatory response, further aiding the remodeling of the cervix in preparation for labor. In this way, IL-8 amplifies the process of cervical ripening by enhancing both the local immune response and ECM degradation.The action of these cytokines is not limited to local cervical remodeling; they also influence uterine activity, thus contributing to the overall progression of labor^[41,45]^. For instance, IL-6 and TNF-α are involved in regulating uterine contractility by stimulating the production of prostaglandins, which are key mediators of uterine contractions^[27]^. The synchronized action of these cytokines ensures that cervical ripening and uterine contractions occur in tandem, facilitating the smooth progression of labor^[16]^. Dysregulation in the balance of these cytokines, such as excessive or insufficient release, can lead to labor complications, including prolonged labor, uterine dysfunction, or failure of cervical ripening^[6]^.

The temporal regulation of cytokine release by monocytes is crucial for ensuring that cervical ripening occurs at the appropriate time during pregnancy^[52]^. Excessive or inappropriate cytokine activity, especially during the later stages of pregnancy, can lead to pathological labor outcomes, including prolonged labor or preterm labor^[35]^. On the other hand, insufficient cytokine production can result in an inadequate inflammatory response, preventing cervical remodeling and delaying the onset of labor^[33]^. A delicate balance between pro-inflammatory and anti-inflammatory cytokines is necessary to ensure optimal cervical ripening and labor progression^[31]^. Disruptions to this balance can significantly impact maternal and neonatal health, highlighting the importance of understanding cytokine regulation in the context of labor^[19]^. Research into the role of monocyte-derived cytokines in cervical ripening has provided valuable insights into the mechanisms underlying labor initiation and progression^[2]^. The identification of key cytokines involved in cervical remodeling and uterine contractility opens the door for potential therapeutic interventions aimed at modulating these inflammatory responses^[1]^. For example, cytokine inhibitors or modulators could be developed to either enhance or suppress the inflammatory response, depending on the clinical scenario^[12]^. In women experiencing prolonged labor, strategies to enhance cytokine activity could help promote cervical ripening and uterine contractions, reducing the need for cesarean delivery^[16]^. Conversely, in cases of preterm labor, therapies that suppress premature cytokine release could help delay labor onset, improving neonatal outcomes^[30]^.

### Prolonged labor pathophysiology

Prolonged labor, also known as labor dystocia, is a condition where the process of childbirth fails to progress normally within the expected timeframe, typically beyond 18–24 hours for a first-time mother and 12–14 hours for women who have previously given birth^[25]^. It is a multifactorial condition involving a complex interplay of maternal, fetal, and uterine factors that prevent the normal progression of labor^[53]^. The pathophysiology of prolonged labor revolves around multiple causes, including uterine dysfunction, abnormal cervical ripening, fetal malposition, and inadequate uterine contractions, with the immune system and inflammatory responses playing a critical role in the process^[54]^. One of the central factors in the pathophysiology of prolonged labor is the improper cervical ripening and dilation^[26]^. The cervix must soften, thin, and dilate to allow for the passage of the fetus through the birth canal^[39]^. This process, known as cervical ripening, is regulated by a combination of hormonal changes and inflammatory responses, particularly the activity of immune cells such as monocytes, macrophages, and neutrophils^[30]^. In prolonged labor, the normal inflammatory cascade that triggers cervical softening and remodeling can be disrupted, leading to a failure of the cervix to properly dilate and efface^[54]^. Monocytes, which are a key component of the innate immune response, are involved in cervical remodeling through the secretion of cytokines and MMPs^[55]^. However, when the function of these immune cells is dysregulated, it can impair cervical ripening, leading to labor stagnation^[19]^.

Uterine dysfunction is another critical factor contributing to prolonged labor^[56]^. The uterus must contract in a coordinated manner to expel the fetus. In cases of prolonged labor, uterine contractions can become ineffective, either due to a lack of coordination or insufficient intensity^[57]^. This uterine dysfunction can arise from a variety of causes, including uterine muscle fatigue, structural abnormalities, or insufficient levels of uterine-stimulating hormones like oxytocin and prostaglandins^[58]^. Inflammatory mediators, particularly cytokines released by immune cells such as monocytes, play a pivotal role in stimulating uterine contractions^[59]^. Disruptions in this inflammatory signaling, such as an imbalance between pro-inflammatory and anti-inflammatory cytokines, can lead to ineffective uterine contractions, contributing to labor dysfunction^[16]^. Fetal factors, such as malposition, fetal macrosomia (larger-than-normal baby), or abnormal presentation, can also contribute to prolonged labor^[56]^. When the fetus is in a less favorable position (e.g., breech or posterior presentation), it can obstruct the normal descent through the birth canal, resulting in delayed or arrested labor^[25]^. These issues often require additional interventions, such as manual manipulation or cesarean delivery^[60]^. The fetal head’s engagement with the cervix and its ability to exert pressure are crucial for cervical dilation and the progression of labor. In cases of fetal malposition, the pressure exerted on the cervix may be insufficient, preventing adequate cervical effacement and dilation^[61]^.

**Figure 2.**
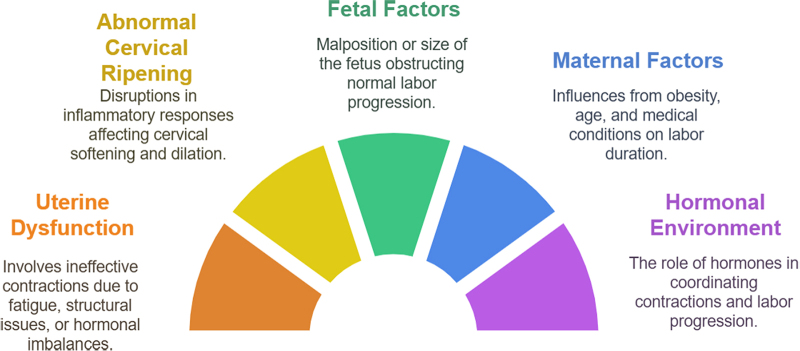
Schematic of pathophysiology in prolonged labor.

Maternal factors also play a significant role in the development of prolonged labor^[57]^. Obesity, age, and pre-existing medical conditions such as diabetes, hypertension, or infections can increase the risk of prolonged labor^[62]^. Obesity, in particular, is associated with chronic low-grade inflammation, which can disrupt normal immune function, impairing the inflammatory response needed for cervical ripening^[63]^. Additionally, maternal infections such as urinary tract infections (UTIs) or bacterial vaginosis (BV) may increase the local inflammatory response, potentially leading to an excessive inflammatory environment in the cervix^[27]^. This heightened inflammation can interfere with the normal cervical remodeling process, further complicating labor progression^[16]^. The hormonal environment plays a crucial role in coordinating the different aspects of labor. The primary hormones involved in labor include estrogen, progesterone, oxytocin, and prostaglandins^[64]^. A delicate balance of these hormones is required for uterine contractions, cervical ripening, and labor progression. Dysregulation in hormonal signaling can contribute to prolonged labor^[65]^. For example, a deficiency in oxytocin, which is responsible for uterine contractions, can result in uterine atony or insufficient contractions, while excessive progesterone levels can inhibit uterine contractions and delay the onset of labor^[66]^. In some cases, prolonged labor may result from psychological factors such as maternal stress or anxiety. Stress hormones like cortisol can influence the immune system and contribute to the inflammatory environment within the cervix^[44]^. Additionally, psychological stress may hinder uterine contractions, further contributing to labor delay^[57]^. Thus, managing maternal stress levels through support, relaxation techniques, and pain management strategies may be beneficial in addressing prolonged labor in certain cases^[26]^, as shown in Figure 2. Figure 2 shows the schematic of pathophysiology in prolonged labor.

### Monocyte dysregulation in prolonged labor

Monocyte dysregulation plays a significant role in the pathophysiology of prolonged labor, a condition in which labor fails to progress despite the presence of uterine contractions^[67]^. Prolonged labor can result from various factors, but immune system dysfunction, particularly the disruption of monocyte and macrophage function, is increasingly recognized as a key contributor to this condition^[1]^. Monocytes, which are a critical component of the innate immune system, normally participate in cervical ripening by secreting inflammatory cytokines, promoting MMP activity, and facilitating cervical remodeling^[68]^. However, when the monocyte response is dysregulated, it can impair the physiological changes needed for cervical softening, effacement, and dilation, ultimately leading to labor stagnation^[69]^. One of the primary mechanisms through which monocyte dysregulation affects labor is through the imbalance of pro-inflammatory cytokines^[55]^. Monocytes are known to secrete a range of cytokines, including IL-1, IL-6, TNF-α, and IL-8, that play a central role in initiating and coordinating cervical ripening^[43]^. These cytokines promote the breakdown of the ECM, enhancing cervical softening, and stimulate prostaglandin production, which induces uterine contractions^[44]^. However, in the case of prolonged labor, an overactive or underactive monocyte response may result in excessive or insufficient cytokine production^[55]^. For instance, excessive TNF-α production can lead to tissue damage and excessive inflammation, inhibiting cervical dilation and causing dysfunctional labor^[1]^. Conversely, insufficient cytokine activity or an inadequate monocyte response can prevent the necessary ECM degradation and cervical remodeling, leading to an inability to achieve full cervical dilation^[44]^.

A key feature of monocyte dysregulation in prolonged labor is the altered recruitment and activation of monocytes at the cervix^[70]^. Normally, chemokines like MCP-1 attract monocytes to the cervix, where they differentiate into macrophages and initiate the inflammatory process that leads to cervical ripening^[71]^. In cases of prolonged labor, this recruitment may be impaired, or monocytes may fail to activate properly upon reaching the cervix^[55]^. This failure to recruit or activate monocytes results in a lack of necessary immune signaling and cytokine production at the cervix, thereby impairing cervical remodeling^[72]^. In some cases, this can be associated with a reduced influx of other immune cells, such as neutrophils, which further exacerbates the failure of the inflammatory cascade needed for effective labor^[73]^. Furthermore, monocyte dysfunction in prolonged labor is often linked to an imbalance between pro-inflammatory and anti-inflammatory mediators^[16]^. Normally, a balance between these two types of mediators is essential to ensure proper immune function during labor. While pro-inflammatory cytokines, like TNF-α and IL-1, stimulate labor progression, anti-inflammatory cytokines, such as IL-10, play a role in resolving inflammation and preventing excessive tissue damage^[43]^. Dysregulation in this balance, such as an overactive pro-inflammatory response or insufficient anti-inflammatory signaling, can result in a state of chronic inflammation, which inhibits normal cervical ripening and impedes labor progress^[60]^. This imbalance could be due to an overproduction of pro-inflammatory mediators or a reduced capacity to produce sufficient anti-inflammatory cytokines, thereby leading to impaired cervical remodeling and prolonged labor^[1]^.

**Figure 3.**
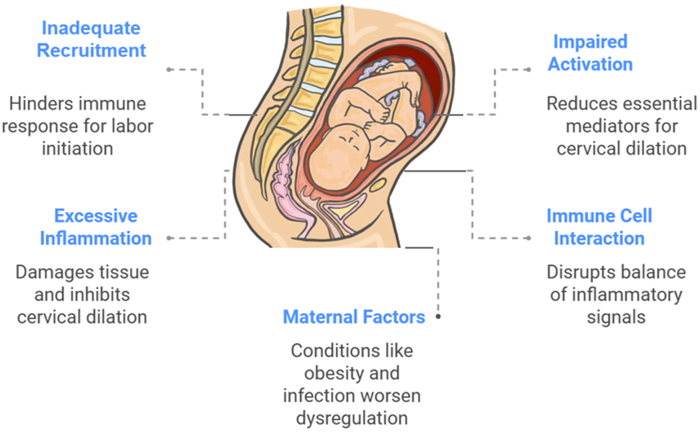
Schematic of monocyte dysregulation in prolonged labor.

The disruption of monocyte function may also contribute to pathological alterations in uterine contractility^[59]^. In a normal labor scenario, monocyte-derived cytokines, particularly IL-6 and TNF-α, promote uterine smooth muscle contraction and increase prostaglandin synthesis, which are essential for the progression of labor^[51]^. However, when monocyte activation is dysfunctional, the resulting cytokine profile may be insufficient to stimulate proper uterine contractility^[23]^. A failure to appropriately activate uterine smooth muscle may lead to ineffective contractions, which is a common feature of prolonged labor^[19]^. Additionally, dysregulated cytokine activity can lead to alterations in the hormonal environment, such as reduced levels of oxytocin or prostaglandins, further contributing to uterine dysfunction and labor arrest^[18]^. Research has shown that maternal factors, such as obesity, infections, or pre-existing inflammatory conditions, can influence monocyte dysregulation and increase the likelihood of prolonged labor^[16]^. In conditions like obesity, the systemic inflammatory environment is often heightened, leading to an exaggerated monocyte response^[12]^. Chronic low-grade inflammation in these individuals may impair the normal recruitment and activation of monocytes at the cervix, disrupting the cervical ripening process^[74]^. Similarly, infections such as UTIs or BV can lead to an overactive immune response, resulting in an excessive release of pro-inflammatory cytokines^[75]^. This heightened inflammatory state can prevent effective cervical dilation and cause labor to stall^[48]^, as shown in Figure 3. Figure 3 shows the schematic of monocyte dysregulation in prolonged labor.

These findings highlight the importance of monitoring maternal health and managing underlying conditions that may contribute to monocyte dysregulation^[72]^. Therapeutic interventions aimed at correcting monocyte dysfunction hold promise for improving the management of prolonged labor^[55]^. For example, the use of cytokine inhibitors or modulators may be beneficial in cases where excessive inflammation is contributing to labor dysfunction^[76]^. By reducing the overproduction of inflammatory cytokines, it may be possible to alleviate the tissue damage and dysfunction that impedes cervical ripening and uterine contractions^[44]^. Conversely, strategies that promote monocyte activation or enhance cytokine production may be useful in cases where monocyte dysfunction leads to insufficient cervical remodeling^[29]^. In such cases, interventions could focus on promoting an adequate inflammatory response to ensure proper labor progression^[19]^. The development of such targeted therapies, however, requires a deeper understanding of the molecular signaling pathways that govern monocyte function during labor^[55]^.

## Implications for therapeutic interventions

Monocyte dysregulation in prolonged labor presents an opportunity for targeted therapeutic interventions aimed at restoring proper immune function and ensuring effective cervical ripening and uterine contractions^[77]^. A comprehensive understanding of how monocytes and their derived cytokines influence the progression of labor can guide the development of novel strategies to manage labor complications^[72]^. Therapeutic interventions that modulate monocyte activity – either by enhancing or inhibiting cytokine production – could potentially improve labor outcomes, particularly for women experiencing prolonged or dysfunctional labor^[72]^. These interventions can be categorized into strategies aimed at modulating cytokine responses, regulating immune cell recruitment, and addressing the underlying causes of monocyte dysfunction^[55]^. One potential approach for managing prolonged labor related to monocyte dysregulation is the use of cytokine inhibitors^[70]^. In cases where an overactive inflammatory response, such as excessive TNF-α or IL-1 production, is hindering cervical ripening and uterine contractility, the administration of cytokine-blocking agents may be beneficial^[50]^. For instance, TNF-α inhibitors, such as infliximab, or IL-1 antagonists could help reduce the excessive inflammatory activity that impedes cervical dilation and uterine smooth muscle function^[50]^. By decreasing the inflammatory burden, these inhibitors could promote a more regulated and effective cervical remodeling process, leading to improved labor progression and potentially reducing the need for cesarean delivery^[35]^. However, careful monitoring would be required to prevent the suppression of necessary immune responses that are critical for normal labor progression^[31]^.

Conversely, in cases where there is insufficient monocyte activation or cytokine production, particularly in women with poor cervical ripening and weak uterine contractions, therapeutic strategies aimed at enhancing immune activation may be more appropriate^[27]^. One such approach could involve the use of cytokine agonists or immune modulators that promote the production of pro-inflammatory cytokines, such as IL-6, IL-8, or TNF-α, at the cervix^[51]^. These mediators would help stimulate the necessary immune responses to facilitate ECM degradation, cervical softening, and increased prostaglandin synthesis^[78]^. Prostaglandins, which are crucial for uterine contractions, could also be enhanced by promoting cytokine signaling pathways that support their production^[31]^. Additionally, therapies aimed at stimulating monocyte differentiation into macrophages or promoting their recruitment to the cervix could help strengthen the local immune response and improve cervical remodeling, accelerating labor progression^[59]^. Another promising therapeutic strategy involves the regulation of monocyte recruitment and activation at the cervix. Chemokine signaling, particularly the recruitment of monocytes to the cervix via MCP-1, is a key factor in initiating the inflammatory response necessary for cervical ripening^[79]^. In cases where monocyte recruitment is impaired, therapies designed to enhance chemokine signaling or monocyte trafficking could help restore proper immune activation at the cervix^[80]^. This could be achieved through the use of chemokine agonists or small molecules that promote the movement of monocytes to the cervix, facilitating cytokine release and ECM remodeling^[81]^. Conversely, in situations where excessive monocyte activation contributes to pathological inflammation, inhibitors of chemokine signaling pathways could be used to prevent the over-recruitment of monocytes, thus reducing the risk of labor dysfunction caused by excessive inflammation^[12,82]^.

Addressing maternal health conditions that contribute to monocyte dysregulation may also serve as a complementary strategy for managing prolonged labor^[74]^. For example, conditions such as obesity, diabetes, or infections can exacerbate systemic inflammation and lead to monocyte dysfunction, further contributing to labor complications^[73]^. By managing these predisposing factors, it may be possible to reduce the risk of monocyte dysregulation and improve the overall inflammatory environment in the cervix^[83,84]^. Interventions such as weight management, control of blood sugar levels, and treatment of infections may help restore normal immune function, promoting timely cervical ripening and reducing the likelihood of prolonged labor^[35]^. Additionally, maternal nutritional support could play a role in modulating the immune response, as certain nutrients, such as omega-3 fatty acids and antioxidants, have been shown to exert anti-inflammatory effects and may help improve monocyte function^[1]^. Finally, the use of non-pharmacological interventions to modulate monocyte and immune cell activity offers an alternative or adjunct to pharmaceutical approaches^[70]^. Techniques such as acupressure, acupuncture, and stress management may help reduce systemic inflammation and promote the proper activation of monocytes during labor^[79]^. Research has suggested that these practices may help balance immune responses, reduce pain, and improve uterine function, potentially reducing the incidence of prolonged labor^[55]^. Additionally, the use of uterine stimulants like oxytocin could help enhance uterine contractions, potentially working synergistically with therapies aimed at regulating the inflammatory response to promote labor progression^[26]^.

## Recommendations

Addressing the multifactorial causes of prolonged labor requires a comprehensive approach that integrates medical, pharmacological, and non-pharmacological strategies to optimize maternal and fetal outcomes. The following recommendations outline key areas for intervention based on a deeper understanding of the pathophysiology of prolonged labor, with an emphasis on managing immune dysregulation, uterine dysfunction, and other contributing factors.

- *Targeted immune modulation*: Given the central role of monocytes and inflammatory cytokines in cervical ripening and uterine contractions, strategies aimed at modulating the immune response could be beneficial. In cases where excessive inflammation is hindering cervical ripening, the use of cytokine inhibitors, such as TNF-α or IL-1 antagonists, may help reduce the inflammatory burden and promote normal cervical dilation. Conversely, in instances of insufficient inflammatory activity, therapies aimed at stimulating cytokine production (such as IL-6 or TNF-α) may encourage proper cervical softening and promote uterine contractions. Targeting specific immune pathways to restore normal immune function at the cervix offers a promising avenue for intervention in prolonged labor.
- *Oxytocin and prostaglandin administration*: Pharmacological agents such as oxytocin and prostaglandins play a crucial role in stimulating uterine contractions and promoting cervical ripening. In cases of uterine atony or insufficient contractions, oxytocin administration can help restore contraction frequency and intensity, facilitating labor progression. Prostaglandins, which also contribute to cervical softening, may be administered to promote cervical ripening in cases of poor effacement or dilation. Timely use of these agents can prevent labor from stalling and may reduce the need for cesarean delivery.
- *Cervical ripening agents*: For women with unfavorable cervical conditions (e.g., a thick or unripe cervix), the use of mechanical or pharmacological cervical ripening agents such as misoprostol, dinoprostone, or Foley catheters can be effective. These agents enhance cervical softening and dilation, helping to initiate or accelerate labor. Monitoring for uterine hyperstimulation and adverse effects is crucial when utilizing these agents, and careful patient selection is necessary to ensure their safety and efficacy.
- *Management of maternal health conditions*: Pre-existing maternal health conditions such as obesity, diabetes, and hypertension significantly increase the risk of prolonged labor. These conditions may contribute to monocyte dysregulation, abnormal inflammatory responses, and uterine dysfunction. Managing these underlying health conditions through lifestyle changes (such as weight management and dietary adjustments) and pharmacological interventions (such as insulin control for diabetic patients) is essential for optimizing labor outcomes. Regular prenatal care that includes monitoring for conditions like gestational diabetes and hypertensive disorders is crucial for identifying and managing complications early.
- *Fetal position and presentation*: Abnormal fetal position, such as breech or occiput posterior, can obstruct the normal course of labor and result in prolonged labor. Assessment of fetal position using ultrasound and physical examination should be conducted early in labor, with interventions such as manual rotation or cesarean delivery considered if malposition is identified. Proper fetal positioning may help ensure effective engagement and descent through the birth canal, reducing the likelihood of labor arrest.
- *Psychological support and stress management*: Psychological factors, such as stress and anxiety, can adversely affect labor progression by influencing uterine contractions and immune responses. Maternal stress can increase cortisol levels, which may disrupt the hormonal and immune balance necessary for effective labor. Providing psychological support, including relaxation techniques, coaching, and a supportive birthing environment, can help reduce stress and enhance labor progression. Ensuring that the laboring woman feels empowered and supported can improve both the physical and emotional aspects of the childbirth process.
- *Non-pharmacological interventions*: Non-pharmacological methods such as acupuncture, acupressure, and massage therapy may provide relief from pain and support labor progression. These techniques may help reduce pain perception, promote uterine contractions, and improve overall comfort during labor. Additionally, practices like movement, positioning, and water immersion may aid in optimal fetal positioning and cervical dilation, potentially reducing the need for medical interventions.
- *Early identification and monitoring*: Early identification of women at risk for prolonged labor, such as those with a history of labor complications or certain maternal risk factors (e.g., age, obesity, or pre-existing conditions), is essential. Close monitoring of labor progression, including assessment of cervical dilation, fetal station, and uterine contraction patterns, can help detect labor abnormalities early. In cases where labor progression is slower than expected, timely interventions – such as the use of oxytocin or other pharmacological agents – should be initiated to prevent prolonged labor from leading to further complications.
- *Education and training for health care providers*: Ongoing education and training for health care providers, including obstetricians, midwives, and labor nurses, are vital for recognizing the signs and symptoms of prolonged labor and implementing evidence-based interventions. Providers should be familiar with the latest research on the role of immune responses in labor, particularly in relation to cytokine modulation and monocyte activity. By staying informed about advancements in the understanding of prolonged labor pathophysiology, health care providers can offer better care and make timely decisions during labor.
- *Research into monocyte function and labor*: Continued research into the role of immune cells, especially monocytes and their cytokine products, in the pathophysiology of labor is crucial. Studies focusing on the molecular and cellular mechanisms underlying cervical ripening, uterine contractions, and the inflammatory process will provide new insights into how these processes can be modulated for better labor outcomes. Further research into potential biomarkers of labor progression could also guide the development of personalized treatment plans for women at risk for prolonged labor.

## Conclusion

Monocyte dysregulation plays a pivotal role in the pathophysiology of prolonged labor, with its influence on cervical ripening, uterine contractility, and the overall inflammatory environment of the cervix. The proper function of monocytes is crucial for the timely progression of labor, as they orchestrate cervical remodeling through the release of cytokines and other immune mediators. When monocyte recruitment, activation, or cytokine production is impaired, it can lead to labor dysfunction, characterized by inadequate cervical dilation, weak uterine contractions, and a failure to progress through the stages of labor. Therapeutic interventions aimed at correcting monocyte dysregulation offer significant potential for improving labor outcomes, particularly for women experiencing prolonged labor. Whether through the inhibition of excessive inflammatory cytokines or the promotion of monocyte activation to restore the necessary inflammatory response, targeted therapies may enhance cervical ripening, uterine contractions, and overall labor progression. Additionally, addressing underlying maternal health conditions and utilizing non-pharmacological interventions may complement pharmacological treatments, providing a holistic approach to managing prolonged labor.

## Data Availability

Not applicable as this a review.

## Research registration unique identifying number (UIN)

Not applicable as this is a review.
